# Adapting form to function: can simulation serve our healthcare system and educational needs?

**DOI:** 10.1186/s41077-018-0067-4

**Published:** 2018-06-14

**Authors:** Andrew Petrosoniak, Ryan Brydges, Lori Nemoy, Douglas M. Campbell

**Affiliations:** 1 Department of Medicine, University of Toronto, Toronto, ON Canada; 2 Allan Waters Family Simulation Centre, St. Michael’s Hospital, Toronto, Canada; 3 Li Ka Shing Knowledge Institute, Toronto, ON Canada; 4 Neonatal Intensive Care Unit, St. Michael’s Hospital, Toronto, ON Canada; 5 Department of Pediatrics, University of Toronto, Toronto, ON Canada

**Keywords:** In situ simulation, Healthcare simulation, Simulation function, Simulation form, Patient safety, Quality improvement, Health professions education

In artistic fields, like graphic design and architecture, designers often experience tension between highlighting a product’s appearance (form) and creating a product that meets the intended purpose (function). Traditionally, simulation educators and champions have valued form: the expensive “high-fidelity” manikin or standardized patient is discussed more often than  the “low-fidelity” task trainer. That preoccupation has jeopardized and de-emphasized the importance of the functions (i.e., educational objectives) the simulation is supposed to address [1–3]. With the availability today of a vast array of simulation techniques and innovations, there is clearly an obligation to use simulation resources wisely and to develop an evidence-base that meaningfully connects simulation form (i.e., simulator, simulation actors/confederates, simulation technique, and location) and simulation function (e.g., for education, for assessment, for evaluating systems).

As an analogy, high-quality research arises first from a focused research question and second from methods chosen to best answer that question. Similarly, we suggest a simulation session must begin with a clear statement of the session’s objective, which ultimately dictates the session’s form. We believe this principle applies to any form of simulation, be it a task trainer in the simulation center or a large-scale multi-disciplinary team-based event in the clinical environment. Considering the latter, while our community has made strides in de-emphasizing “fidelity” and is focusing on educational objectives in the simulation center, we wonder if the same thinking applies when conducting simulations in the workplace, commonly referred to as in situ simulation (ISS). A recent paper proposes shifting away from typical simulation descriptors (e.g., high-fidelity) to using a more applicable term, translational simulation, to highlight the importance of a functional connection with healthcare priorities and patient outcomes [4].

We agree with Posner et al. [5] that the hallmark of ISS is that it enables the study of “how the clinical environment responds in its natural state, including the personnel, equipment, and systems responsible for care in that environment.” Observing team members in their typical role using their actual equipment and following their processes likely supports a systems-thinking approach not entirely realized with simulation at off-site centers [6]. Numerous studies support applying ISS to identify latent safety threats (LSTs) [7], to test newly designed clinical environments [8], and to teach and practice communication and teamwork skills [9]. Educators often use ISS as a crash test for personnel, equipment, and systems, offering important insights into patient safety threats and potential mitigation strategies. Here, the *function* is to improve patient safety, and the *form* making it possible is ISS. Despite this potential, however, the literature still lacks evidence to help educators and administrators decide which forms of ISS to implement and which functions ISS best serves [10].

As we strive to support healthcare professionals in continuous, lifelong learning, we need evidence to clarify which simulation environment allows us to achieve our specific objectives effectively and efficiently. In producing that evidence, we discourage comparing ISS to a center-based simulation, as the two represent different forms and functions. Instead, we encourage work that tests and delineates the boundaries of function for all forms of simulation (e.g., take-home simulators). For instance, simulation in the workplace is itself a multi-faceted modality which can take two arguably disparate forms: (1) in situ simulation, where scenarios unfold in the actual clinical environment with the actual clinical equipment and (2) *on site* simulation (OSS), where scenarios take place in the workplace, yet somewhere “aside” like a spare or unused patient room with or without the actual clinical equipment from the unit. ISS scenarios enable objectives like testing of equipment, systems, and spaces, whereas OSS scenarios enable functions like reinforcing skills and educational outcomes without removing staff from the familiarity and convenience of the workplace [5]. Their forms differ slightly, while their functions can differ dramatically [4].

In education, we tend to follow cycles of recreating the same old problems in new surroundings. “Innovators” digitized textbooks and called them e-learning, which now prompts a chuckle. Similarly, we see the possibility that educators will simply transport the functions for which center-based simulation is ideal into ISS scenarios. While ISS appears to have a strong footing in identifying patient safety concerns and systems issues, the evidence for superiority in learning outcomes is far less convincing [6, 11]. Hence, it may be that in thinking about the optimal use of ISS, we must think about the unit of analysis. Perhaps ISS is ill-suited for individual-level, skill-based objectives requiring deliberate and regular practice, yet ISS might be fit-for-purpose to study teams, clinical units, and institutional level quality improvement initiatives [12]. We believe ISS represents a valuable tool for clinicians, educators, and patient safety experts when used appropriately, always using the principle that form follows function.

Ultimately, an integrated system of simulation across settings, from center-based to OSS to ISS, will help us align institutional-, unit-, team-, and individual-level goals. Yet, our community remains in a perpetually early state when it comes to clarifying and testing the boundaries of how each form of simulation might function best. Despite the increasingly widespread use of ISS in different settings, for example, our understanding of the mechanisms through which ISS can increase the reliability and resilience of organizational activities remains under-developed [10]. Authors of a remarkably high number of papers laud the capacity for ISS to identify LSTs, provide lists of those LSTs (sometimes in a prioritized order), and then leave us to wonder what happened: How were they addressed? Has the organization realized patient safety benefits? What are the lessons learned of actually tackling that list, beyond writing it on a page? Let us move beyond rhetoric, let us move beyond descriptive studies, and let us begin the hard work of collecting empirical evidence for how and why ISS, OSS, center-based simulation, and other technology-enabled activities can function in an integrated system to produce adaptive learners, teams, and systems. In establishing the art and science of simulation, let us remember the lessons from professional designers: meaningfully connect simulation form and simulation function. Doing so will help clarify the optimal use of all forms of simulation - from paper-based cases to full-scale emergency preparedness training with many simulated patients, family members, and care providers.

## Abbreviations

ISS: In situ simulation
OSS: On-site simulation
